# Enteric duplication cyst as a lead point for intrauterine segmental volvulus resulting in type IIIa ileal atresia: a neonatal case report and focused literature review

**DOI:** 10.3389/fped.2026.1888442

**Published:** 2026-08-10

**Authors:** Mariam Marzouki, Malek Mezni, Yosra Ben Ahmed, Wiem Hammouda, Rim Ben Aziza, Imen Ben Ismail, Said Jlidi, Aya Khemir, Imen Abess

**Affiliations:** 1 Department of Pediatric Surgery “Hichem Said”, Children’s Hospital of Tunis, University Tunis El Manar, Tunis, Tunisia; 2 Neonatal Intensive Care Unit, Center of Maternity and Neonatology of Tunis, University Tunis El Manar, Tunis, Tunisia; 3 Department of Visceral and Digestive Surgery, Traumatology and Great Burns Center Ben Arous, University of Tunis El Manar, Tunis, Tunisia; 4 Pathology Department, Salah Azaiez Institute, University of Tunis El Manar, Tunis, Tunisia

**Keywords:** case report, enteric duplication cyst, ileal atresia, segmental volvulus, vascular pathogenesis

## Abstract

**Background:**

Jejunoileal atresia is most commonly attributed to intrauterine mesenteric vascular accidents. Both segmental volvulus and enteric duplication cysts are recognized yet uncommon causes of fetal vascular compromise capable of inducing intestinal ischemia. The simultaneous occurrence of ileal atresia, segmental volvulus, and enteric duplication cyst in a single neonate is exceedingly rare and offers valuable insight into the vascular pathogenesis of intestinal atresia.

**Case presentation:**

We report the case of a male term neonate admitted eleven hours after birth for bilious vomiting and progressive abdominal distension. Plain abdominal radiography revealed multiple air-fluid levels, and contrast enema demonstrated a microcolon with characteristic “string-of-beads” filling defects in the distal ileum consistent with meconium pellets. Exploratory laparotomy identified a type IIIa distal ileal atresia located 20 cm from the ileocecal valve, associated with a segmental volvulus twisting around a 3 cm cystic enteric duplication. The volvulated bowel segment, duplication cyst, and dilated proximal ileum were resected, and a primary end-to-end ileoileal anastomosis was performed. Histopathology confirmed cystic ileal duplication with bilateral ileal atresia at the resection margins. Despite an initially stable postoperative course, the infant developed severe bronchiolitis with respiratory failure and died on postoperative day 10.

**Conclusion:**

This rare neonatal triad supports the hypothesis that enteric duplication cysts may act as intrauterine lead points for segmental volvulus, triggering mesenteric vascular compromise and secondary ileal atresia. Any neonate presenting with distal bowel obstruction and microcolon should prompt consideration of complex intrauterine vascular events. Prompt surgical exploration remains essential to maximize intestinal preservation and improve outcomes.

## Introduction

Jejunoileal atresia is one of the most common causes of neonatal intestinal obstruction, with an estimated incidence of 1 in 2,000 to 5,000 live births (1, 2). It results from an intrauterine mesenteric vascular insult causing ischemic necrosis of the affected bowel segment, a mechanism first established by Louw and Barnard (3). Recognized causes include volvulus, intussusception, internal herniation, mesenteric bands, and vitelline vascular anomalies (4–7), among which segmental volvulus without malrotation is increasingly recognized as a mechanism for secondary ileal atresia (5, 8). Enteric duplication cysts are rare congenital malformations, most often involving the ileum, that may act as lead points for intrauterine volvulus and secondary ischemic bowel injury (9–11).

The simultaneous occurrence of ileal atresia, segmental volvulus, and enteric duplication cyst in a single patient is exceptionally rare. We report such a case in a term male neonate and discuss the pathophysiological sequence linking these three lesions and review the relevant literature.

## Patient information

A term male neonate was referred to our department 11 h after birth for bilious vomiting and progressive abdominal distension. The antenatal history was notable for gestational diabetes and polyhydramnios, without consanguinity, relevant family history, or prenatal diagnosis of intestinal obstruction. Feeding intolerance was present from birth.

## Clinical findings

The infant was hemodynamically stable and afebrile. The abdomen was diffusely distended, soft, and non-tender, without a palpable mass or wall erythema. Hernial orifices and anus were normal. No dysmorphic features were noted. Rectal tube decompression yielded no output.

## Diagnostic assessment

Laboratory tests were unremarkable. Plain radiography showed multiple central air-fluid levels without pneumoperitoneum. Ultrasonography revealed dilated proximal small bowel loops (up to 18 mm) with thickened walls and collapsed, echogenic distal loops suggestive of inspissated meconium; Doppler mesenteric vessel orientation was normal, without free fluid. Water-soluble contrast enema showed a microcolon with minimal progression into the terminal ileum and multiple “string-of-beads” filling defects in the distal ileum, consistent with meconium pellets.

These findings favored a distal obstructive lesion of prenatal origin over malrotation-volvulus, given the normal mesenteric Doppler pattern; the differential included jejunoileal atresia, meconium ileus, and Hirschsprung disease. Prenatal ultrasonography had shown polyhydramnios only, without bowel dilatation or a cystic lesion. The associated duplication cyst and segmental volvulus were not identified preoperatively. Type IIIa distal ileal atresia with segmental volvulus around a cystic enteric duplication was diagnosed at surgery and confirmed histologically.

## Therapeutic intervention

**Figure 1:**
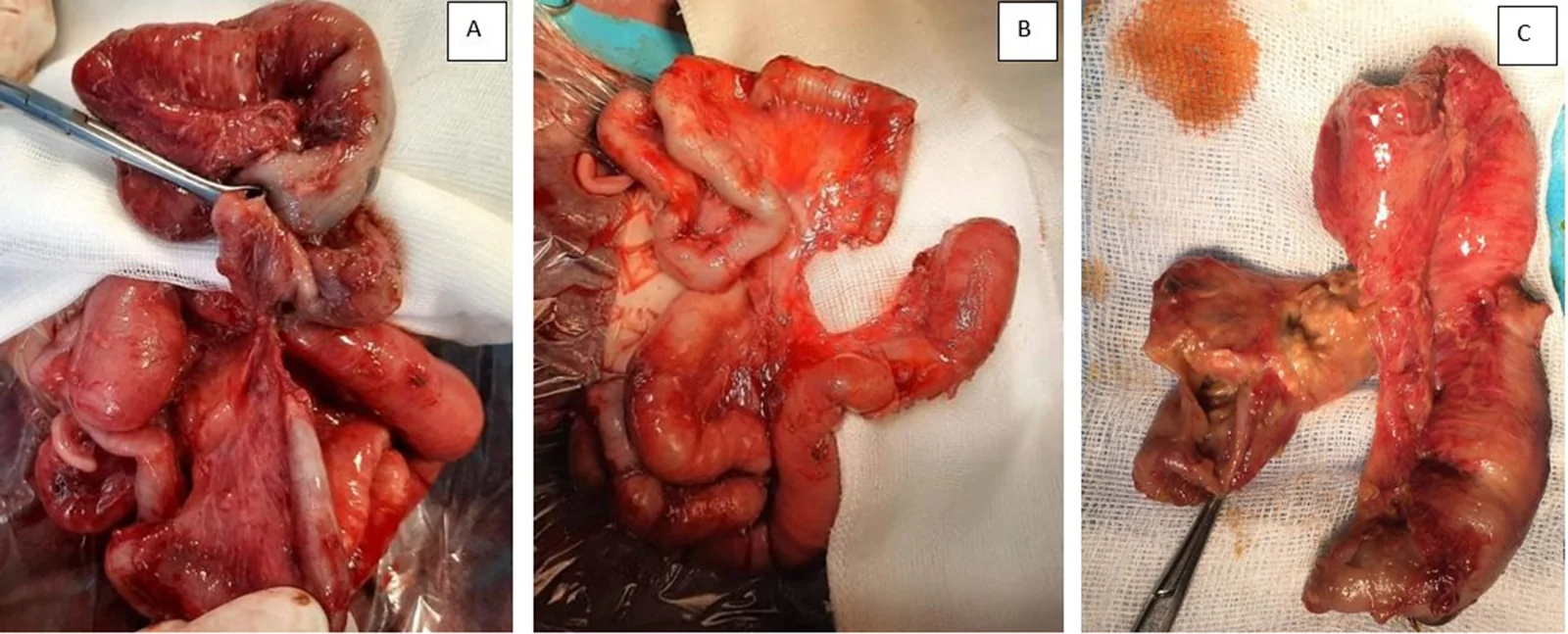
**(A)** Intraoperative view showing segmental volvulus between the two atretic ileal segments. **(B)** Intraoperative view after resection of the volvulated loop and duplication cyst, demonstrating a Type IIIa small bowel atresia. **(C)** Resected specimen showing the enteric duplication cyst attached to the volvulated ileal segment.

Persistent obstruction with bilious nasogastric drainage prompted exploratory laparotomy via a transumbilical midline incision. A type IIIa distal ileal atresia was found 20 cm proximal to the ileocecal valve, with a segmental volvulus involving a 3-cm cystic enteric duplication interposed between the atretic ends (Figure 1A); the proximal bowel was dilated, edematous, and congested, while the distal bowel and colon were collapsed and meconium-filled. No malrotation was present. The volvulated segment, duplication cyst, and 7 cm of dilated proximal ileum were resected (Figures 1B,C), the distal bowel was irrigated, and a primary end-to-end ileoileal anastomosis was performed, leaving approximately 60 cm of small bowel.

## Follow-up and outcomes

**Figure 2:**
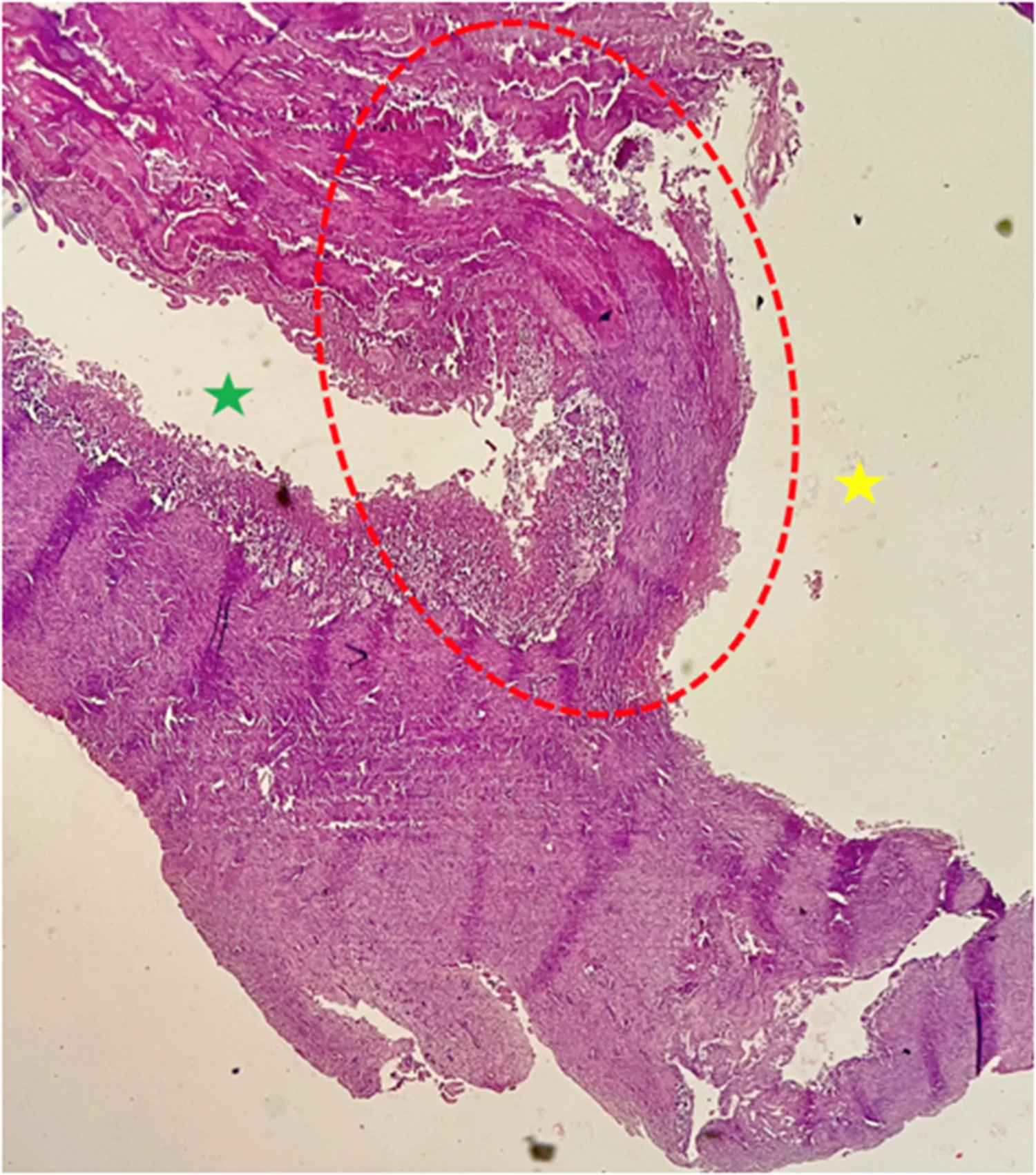
A hematoxylin and eosin stained section at low magnification (x2) showing a tissue sample passing through the septum (in the center; red circle) that separates the cystic cavity (on the right; yellow star) from the lumen of the small intestine (on the left; green star). Both sides of this septum are lined with a small intestine mucosa that is largely denuded (due to poor fixation). This layer is backed on either side by muscularis propria in the septal wall.

**Figure 3:**
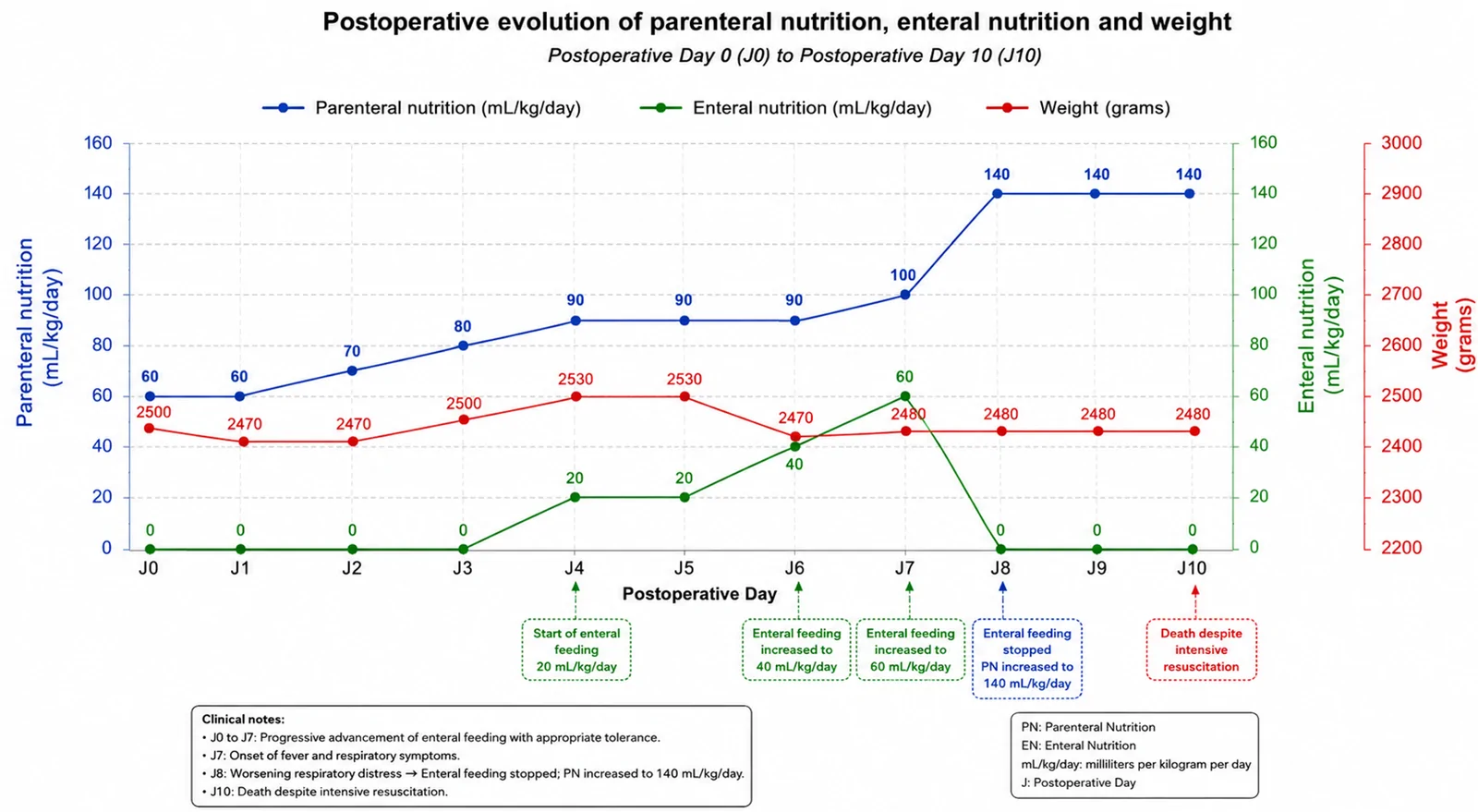
Postoperative evolution of parenteral nutrition, enteral nutrition and weight.

Histopathology confirmed a cystic enteric duplication lined by intestinal mucosa with a smooth muscle wall (Figure 2), and bilateral ileal atresia at the resection margins. Parenteral nutrition was started at 60 mL/kg/day, increasing to 90 mL/kg/day by postoperative day (POD) 4; enteral feeding began on POD 4 and advanced to 60 mL/kg/day by POD 7, with preserved transit but modest weight gain and liquid stools (Figure 3), likely reflecting adaptation to the short residual bowel. On POD 7 the infant developed fever, respiratory distress, and systemic inflammation. Serial abdominal examinations showed a soft, non-distended abdomen without peritoneal signs, and the surgical wound remained unremarkable. Abdominal ultrasonography revealed no intra-abdominal collection, bowel dilatation, or other findings suggestive of an anastomotic complication. Respiratory status progressively worsened despite discontinuation of enteral feeding, increased parenteral support, and intensive care including mechanical ventilation, and the infant died on POD 10. Given the absence of surgical complications, a severe respiratory infection—most likely acute bronchiolitis—was considered the probable cause of death.

Clinical timeline of the reported case is provided in Table 1.

**Table 1:** Clinical timeline of the reported case.

Time point	Clinical event
Prenatal period	Polyhydramnios and gestational diabetes identified on antenatal follow-up
Birth	Term male neonate delivered vaginally; no anomalies detected at birth
11 h of life	Onset of bilious vomiting and progressive abdominal distension; referral to pediatric surgery
Admission (Day 0)	Abdominal radiography, ultrasonography, and contrast enema performed
Day 1	Exploratory laparotomy: type IIIa ileal atresia with segmental volvulus around a cystic enteric duplication identified and resected; primary ileoileal anastomosis performed
Postoperative Days 1–6	Progressive enteral and parenteral nutrition; persistent liquid stools and inadequate weight gain
Postoperative day 7	Development of fever, respiratory symptoms, and biological inflammatory syndrome. Abdominal examinations remained unremarkable. Abdominal ultrasonography showed no evidence of anastomotic or intra-abdominal complications.
Postoperative Days 8–10	Severe hypoxemic respiratory failure requiring intensive care support and invasive mechanical ventilation.
Postoperative Day 10	Death despite maximal resuscitative efforts. Severe respiratory infection considered the most likely cause of death.

## Discussion

Jejunoileal atresia is understood to result from intrauterine mesenteric vascular compromise occurring after normal intestinal organogenesis (3). The foundational experiments of Louw and Barnard — showing that surgical interruption of mesenteric blood flow in fetal dogs reliably reproduces the full spectrum of intestinal atresia seen clinically — established the vascular accident hypothesis that still holds today (3).

Over the following decades, several intrauterine mechanisms capable of generating focal mesenteric ischemia have been documented, including volvulus, intussusception, internal herniation, constrictive bands, and mesenteric vascular anomalies (4–7). Among them, segmental volvulus without malrotation is uncommon, though cases of secondary jejunoileal atresia attributed to this mechanism are being reported with growing frequency in the neonatal literature (5, 8, 12).

Enteric duplication cysts occur in approximately 1 in 4,500 to 5,000 live births (9). They are defined by a gastrointestinal mucosal lining, a smooth muscle wall, and a shared common wall with the adjacent digestive tract. The ileum is the most frequently involved segment. Their clinical presentation is variable and includes bowel obstruction, gastrointestinal bleeding, a palpable abdominal mass, perforation, or, less commonly, torsion-related ischemic injury (9–11).

The mechanism by which a duplication cyst acts as a fixed intrauterine lead point for segmental volvulus, leading to mesenteric ischemia and secondary intestinal atresia, has been described in isolated neonatal case reports. Mirza et al. reported a neonate with intestinal atresia caused by volvulus twisting around an enteric duplication cyst, and comparable cases have been documented in the context of vitellointestinal remnants and mesenteric defects (4, 6–8, 10, 11, 13). The cases most directly relevant to this association are summarized in Table 2.

**Table 2:** Published reports of intestinal atresia associated with segmental volvulus and intrauterine lead points*.*

Author	Year	Associated lesion	Volvulus	Atresia type	Proposed mechanism
Black et al. (4)	1994	Mesenteric defect	Yes		Mesenteric vascular compromise
Mohammad et al. (6)	2016		Yes	Ileal atresia	Vascular insufficiency
Mirza et al. (10)	2018	Enteric duplication cyst	Yes	Ileal atresia, jejunal atresia	Volvulus around duplication cyst
Deshpande et al. (7)	2020	Vitellointestinal remnants	Yes	Ileal atresia	Intrauterine vascular insult
Toyama et al. (8)	2023	Dilated bowel torsion	Yes	Ileal atresia	Fetal bowel ischemia
Mili et al. (13)	2023	Mesenteric defect	Yes	Distal ileal atresia	Segmental vascular compromise
Present case	2025	Enteric duplication cyst	Yes	Type IIIa ileal atresia	Duplication cyst → volvulus → ischemia → atresia

What sets this case apart is the simultaneous intraoperative identification of all three lesions in anatomical continuity: the segmental volvulus was centered directly around the duplication cyst and situated between the two atretic bowel ends. These findings provide coherent anatomical support for a stepwise pathophysiological sequence: enteric duplication cyst → intrauterine segmental volvulus → mesenteric vascular compromise → type IIIa ileal atresia.

Neonates with ileal atresia typically present with signs of distal intestinal obstruction, including bilious vomiting and abdominal distension. Palpable abdominal masses may be noted if associated enteric duplications are large. Plain radiographs often show multiple air-fluid levels suggestive of obstruction (12, 14). Contrast enema typically confirms a microcolon, reflecting the absence of antegrade intestinal transit during fetal life as observed in our patient (2).

In our patient, the preoperative identification of the volvulus and duplication cyst was not possible, illustrating the diagnostic limitations inherent to this entity.

Prenatal MRI has been proposed as a tool capable of improving the detection of intrauterine volvulus and associated intestinal complications, though its role remains investigational (15).

The surgical management of jejunoileal atresia is guided by two core principles: preserving as much bowel length as possible while resecting all non-viable or structurally compromised segments (16). When bowel ends are healthy and well-perfused, primary anastomosis following resection remains the standard approach. In our patient, the intraoperative appearance of the volvulated segment left no alternative to resection.

Neonatal segmental volvulus without malrotation tends to carry a more favorable prognosis than midgut volvulus, as ischemic damage is typically more localized and the extent of bowel loss more limited. However, delayed diagnosis, extensive intestinal resection, or the presence of additional congenital anomalies may substantially worsen outcomes (17–19). In our case, the fatal outcome was attributable to an unrelated intercurrent respiratory complication rather than a direct consequence of the intestinal surgery.

The main strength of this report is the intraoperative identification of all three lesions simultaneously and in anatomical continuity, offering direct evidence in support of the proposed vascular pathogenic sequence. Histopathological confirmation further strengthens this conclusion. The main limitation is the inherent constraint of single-case reporting, which precludes generalization. The lack of prenatal imaging evidence of either the volvulus or the duplication cyst further illustrates the challenge of diagnosing this rare entity prior to surgical exploration.

The association of ileal atresia, segmental volvulus, and enteric duplication cyst in a single neonate is rare. This case adds anatomical and histopathological evidence that an enteric duplication cyst can act as a lead point for intrauterine segmental volvulus, ultimately causing mesenteric vascular compromise and secondary ileal atresia.

In neonates presenting with persistent distal bowel obstruction and microcolon, surgeons should keep in mind the possibility of complex intrauterine vascular lesions associated with congenital anomalies. Early surgical exploration, careful bowel-preserving resection, and primary anastomosis remain the foundation of management and offer the best chance of a good intestinal outcome**.**

## Patient perspective

The patient's parents expressed their gratitude for the care and dedication of the medical team throughout their son's hospitalization. They acknowledged the severity and complexity of the neonatal intestinal condition, as well as the exceptional nature of the associated complications. Despite the tragic outcome, they expressed their wish that this case might contribute to advancing knowledge and improving care for future patients with similar conditions.

## Data Availability

The original contributions presented in the study are included in the article/Supplementary Material, further inquiries can be directed to the corresponding author.
